# Children and Crime

**Published:** 1921-04-30

**Authors:** 


### Children and Crime.

Considerable publicity has been given to a resolution passed by the Council of the Institute of Journalists, by which, "in the interests of public morality and the training of future citizens," all newspapers are urged "to withhold the names of juvenile offenders tried or convicted in the Children's Courts, as well as those of children who are innocently involved in criminal cases." Many newspapers have already adopted this principle, which costs nothing and in no way diminishes the value of any reports of the cases that it may be desirable to publish. The surprising thing is that a single newspaper should continue to give names in these proceedings when there have been so many protests, based on such solid grounds. The resolution passed by the Institute of Journalists should make it still more difficult for the offending newspapers to persist in the practice.

